# Psychological and Behavioral Changes during Confinement in a 520-Day Simulated Interplanetary Mission to Mars

**DOI:** 10.1371/journal.pone.0093298

**Published:** 2014-03-27

**Authors:** Mathias Basner, David F. Dinges, Daniel J. Mollicone, Igor Savelev, Adrian J. Ecker, Adrian Di Antonio, Christopher W. Jones, Eric C. Hyder, Kevin Kan, Boris V. Morukov, Jeffrey P. Sutton

**Affiliations:** 1 Department of Psychiatry, University of Pennsylvania Perelman School of Medicine, Philadelphia, Pennsylvania, United States of America; 2 Pulsar Informatics, Philadelphia, Pennsylvania, United States of America; 3 Wyle, Houston, Texas, United States of America; 4 National Space Biomedical Research Institute, Houston, Texas, United States of America; 5 Institute for Biomedical Problems, Moscow, Russia; 6 Center for Space Medicine and Department of Medicine, Baylor College of Medicine, Houston, Texas, United States of America; Medical University of Graz, Austria

## Abstract

Behavioral health risks are among the most serious and difficult to mitigate risks of confinement in space craft during long-duration space exploration missions. We report on behavioral and psychological reactions of a multinational crew of 6 healthy males confined in a 550 m^3^ chamber for 520 days during the first Earth-based, high-fidelity simulated mission to Mars. Rest-activity of crewmembers was objectively measured throughout the mission with wrist-worn actigraphs. Once weekly throughout the mission crewmembers completed the Beck Depression Inventory-II (BDI-II), Profile of Moods State short form (POMS), conflict questionnaire, the Psychomotor Vigilance Test (PVT-B), and series of visual analogue scales on stress and fatigue. We observed substantial inter-individual differences in the behavioral responses of crewmembers to the prolonged mission confinement and isolation. The crewmember with the highest average POMS total mood disturbance score throughout the mission also reported symptoms of depression in 93% of mission weeks, which reached mild-to-moderate levels in >10% of mission weeks. Conflicts with mission control were reported five times more often than conflicts among crewmembers. Two crewmembers who had the highest ratings of stress and physical exhaustion accounted for 85% of the perceived conflicts. One of them developed a persistent sleep onset insomnia with ratings of poor sleep quality, which resulted in chronic partial sleep deprivation, elevated ratings of daytime tiredness, and frequent deficits in behavioral alertness. Sleep-wake timing was altered in two other crewmembers, beginning in the first few months of the mission and persisting throughout. Two crewmembers showed neither behavioral disturbances nor reports of psychological distress during the 17-month period of mission confinement. These results highlight the importance of identifying behavioral, psychological, and biological markers of characteristics that predispose prospective crewmembers to both effective and ineffective behavioral reactions during the confinement of prolonged spaceflight, to inform crew selection, training, and individualized countermeasures.

## Introduction

With the completion of the International Space Station (ISS) and expanding multinational involvement in space flight, the first human interplanetary mission to Mars is anticipated during this century. Using conventional propulsion and accounting for celestial mechanics, a round trip of 520 days is a standard reference mission. This timeframe is well beyond the duration astronauts and cosmonauts have remained confined either in a spacecraft or in a high-fidelity spaceflight simulation on Earth. Current missions on ISS are 6 months in duration. Only 4 people have spent more than 1 year in a spacecraft, with the record for continuous confinement set by Valery Polyakov at 437 days on Mir. The longest Earth-based space flight simulation (SFINCSS-99) involved 4 Russians confined in connected hyperbaric chambers for 240 consecutive days (one crewmember was confined for 263 days) [1]–[3]. Due to communication delays, a Mars mission will also require greater crew autonomy than currently experienced in spaceflight [4]. A U.S. National Academies report on astronaut care for exploration missions concluded that behavioral and mental health issues will be increasingly important during such missions, which will likely involve a crew varying in social and cultural backgrounds. The report urged research focus on the behavior of astronauts in extreme, isolated microenvironments such as inside spacecraft [5]. NASA's recent evidence-based review of the behavioral health risks to crew and mission success during exploration space flight concluded they were among the most serious risks to such missions [6], a view shared by the Aerospace Medical Association [7]. According to NASA [6], “anecdotal and empirical evidence indicates that the likelihood of a behavioral condition or psychiatric disorder occurring increases with the length of a mission” and “while behavioral conditions or psychiatric disorders might not immediately and directly threaten mission success, such conditions can, and do, adversely impact individual and crew health, welfare, and performance, thus indirectly affecting mission success.”

There is a critical need to predict the time course, magnitude, and individual variability in behavioral, cognitive, affective and interpersonal reactions of space explorers during long-duration missions. Accurate prediction will inform strategies for crew selection, spacecraft habitability requirements, and behavioral health countermeasures needed for interplanetary missions. High-fidelity simulated space flight has paramount importance in providing data on crew behavioral changes during prolonged confinement and isolation. However, the ecological validity of the simulation depends heavily upon the extent to which it instantiates elements relevant to crew behavior during prolonged confinement in space. These include crew characteristics and size, habitat and habitability, isolation from Earth's light-dark cycles and weather, mission duration and realistic mission operations, flight simulation with mission controllers, communication delays inherent in interplanetary missions, limited consumable resources, and attention from media and the public.

Antarctic winter-over conditions require groups of subjects to spend prolonged periods of time in confinement and isolation, and they share some of the other environmental and psychosocial stressors inherent to exploration-type space missions (e.g., monotony, threat-to-life, restricted consumables, non-24 h light-dark cycles). They are used by several space agencies as space analog environments. However, these winter-over analogs usually do not extend beyond one year, they do not have a space mission context, and crew composition and size may not generalize to astronauts on long-duration space missions. The greater the fidelity an analog environment has to prolonged space flight, the greater the opportunity to identify the manner in which behavioral health may be affected by prolonged space missions.

Here we report on the behavioral and psychological effects on a 6-person multinational, culturally diverse crew comparable to space fliers, who were participating in the first high-fidelity simulated 520-day mission to Mars. The simulation was developed and operated by the Institute for Biomedical Problems (IBMP) of the Russian Academy of Sciences. We hypothesized that behavioral and psychosocial responses to the prolonged period of confinement, isolation, and space operational requirements, would change systematically with time in mission and related to mission events (e.g., the mid-mission simulated Mars landing). However, due to the uniqueness and unprecedented duration of this simulation, we made no specific hypotheses related to the direction and duration of any systematic trend, but rather formulated our null hypothesis more neutral as “no difference in responses related to time in mission.” Due to the diverse cultural and educational backgrounds of the crew, we expected inter-individual differences in the way individual crewmembers coped with the prolonged period of confinement and isolation.

## Materials and Methods

### 2.1 Subjects and protocol

The State Scientific Center of the Russian Federation –IBMP of the Russian Academy of Sciences performed the Mars 500 project at the IBMP in Moscow, which consisted of three isolation studies with six crewmembers each: a 14-day pilot study (completed in November 2007), a 105-day pilot study (completed in July 2009), and the main 520-day study simulating a mission to Mars (completed in November 2011), which is the focus of this manuscript.

The high fidelity of the simulation to actual spaceflight was reflected in the following features of the experiment: (i) a multinational crew of N = 6 healthy adult male volunteers selected by the Russian Federation (N = 3), the European Space Agency (N = 2), and the China National Space Administration (N = 1), who were trained together and who were similar in age (average age at hatch closing 32 years, range 27–38), careers, and education (e.g., engineers, physicians, military backgrounds) to astronauts/cosmonauts living on the ISS; (ii) 520 consecutive days of confinement (3 June 2010 to 4 November 2011) in a 550 m^3^ pressurized facility with a volume and configuration comparable to a spacecraft with interconnected habitable modules; (iii) facility modules equipped with life support systems and an artificial atmospheric environment at normal barometric pressure; (iv) activities that simulated aspects of the International Space Station with daily maintenance work, scientific experiments, and exercise; (v) isolation from Earth's daily environmental light-dark cycles, temperatures and seasonal conditions; (vi) a realistic Mars flight simulation based in orbital mechanics and under the direction of mission controllers, with a 30-day Mars orbiting phase (between mission days 244 and 273) and 3 of the 6 crewmembers simulating egresses on the Martian surface (between mission days 257 and 265); (vii) work throughout the 520-day mission that included both routine and simulated emergency events; (viii) changes in communication modes and time delays between mission days 54 and 470 that would occur in transit to and from Mars; (ix) limited consumable resources (food and water); and (x) the crew awareness of frequent publicity of the mission by media and the public. Thus, Mars 520 had many essential features of an isolated and confined environment (ICE) that had the fidelity necessary to study behavioral and psychological reactions to prolonged space flight.

The crew lived on a 5-day work cycle, with two days off, except for simulation of special situations (e.g., emergencies). For the whole mission operations were organized around 24-h clock time. A typical workday would start with personal hygiene and breakfast at 8:00 followed by operative work (including facility inspection), operative meetings, and the preparation of scientific experiments. After lunch (served between 13:30 and 14:30), the crews performed the scientific experiments and exercised until supper was served at ca. 19:30. The rest of the evening could be used for personal time. A total of 91 experiments in the areas of physiology (N = 20), psychology (N = 21), biochemistry, immunology, and biology (N = 34), microbiology (N = 8), and operations and technology (N = 8) were performed during the 520 days of the mission. Not all of the experiments required the crew's active participation. Sampling frequency differed between experiments and ranged from three times during the mission to continuously throughout the mission, with most of the studies sampling data on a regular but discontinuous basis (e.g., once every month).

The study was approved by the Institutional Review Board of the University of Pennsylvania, Philadelphia, Pennsylvania, USA. Prior to the start of the study, all subjects signed written informed consent forms. They were compensated for their participation in the study, and they were free to discontinue the study at any time. The crewmembers revealed their identities before, during and after the simulation. To ensure confidentiality in this manuscript, results were de-identified (i.e., crewmembers were randomly assigned English alphabetic letters *a–f*) and no data were reported relative to crewmembers' nationalities, ages, professions, or roles in the mission.

### 2.2 Experimental procedures and measurements

#### 2.2.1 Psychological measures

All instructions and subjective rating scales were translated and displayed in Russian for the three Russian participants, and in English for the non-Russian participants.

2.2.1.1 Social Desirability Scale 17 (SDS-17): Once during the two weeks prior to hatch closing, each crewmember filled out the Social Desirability Scale 17 (SDS-17), which measured social desirability bias [8]. The latter is defined as “distorting one's self-presentation to make a favorable impression upon others.” Social desirability may represent, among others, internalization of cultural values, the expression of ongoing personality traits, or an overly favorable self-evaluation [9]. The SDS-17 is composed of 16 true–false items (one item was dropped from the final scale), six of them reverse keyed. A score of 16 represents maximal distortion of one's self representation to make a favorable impression.

2.2.1.2 Visual Analog Scales (VAS): Immediately prior to and/or following each PVT-B test bout, crewmembers filled out several computerized questionnaires and rating scales. Crewmembers indicated their current status on 100 mm visual analogue scales (VAS) with the following binary anchors: happy – unhappy; healthy – sick; energetic – physically exhausted; mentally sharp – mentally fatigued; not stressed – very stressed; fresh/ready to go – tired; good sleep quality – poor sleep quality (morning only); and high workload – low workload (evening only).

2.2.1.3 Profile of Mood States - Short Form (POMS-SF): Crewmembers completed the POMS-SF [10], [11] in the morning once each week. POMS-SF is a measure of psychological distress in a variety of healthy, physically ill, and psychiatric populations. It consists of a list of 37 adjectives. Crewmembers had to indicate the degree to which each adjective described themselves at the moment they took the test using a 5-point Likert format. Standard scoring of the POMS yields a global distress score referred to as Total Mood Disturbance as well as scores for six subscales: Fatigue-Inertia, Vigor-Activity, Tension-Anxiety, Depression-Dejection, Anger-Hostility, and Confusion-Bewilderment.

2.2.1.4 Beck Depression Inventory (BDI-II): Once a week in the evening, crewmembers completed the modified Beck Depression Inventory (BDI-II) [12], a 21-question multiple-choice self-report inventory for measuring the severity of depression. The BDI-II was scored by summing the highest ratings for each item. Each item is rated on a 4-point scale ranging from 0 to 3, and the total scores can range from 0 to 63. The BDI-II can be separated in an 8-item affective subscale (pessimism, past failures, guilt feelings, punishment feelings, self-dislike, self-criticalness, suicidal thoughts or wishes, and worthlessness) and a 13-item somatic subscale (sadness, loss of pleasure, crying, agitation, loss of interest, indecisiveness, loss of energy, change in sleep patterns, irritability, change in appetite, concentration difficulties, tiredness and/or fatigue, and loss of interest in sex). Crewmembers were asked to indicate how they felt during the past week. The question on suicidal thoughts was removed from the BDI-II for reasons of cultural sensitivity, reducing the number of items to 20 and the maximum score from 63 to 60.

2.2.1.5 Conflict Questionnaire (CQ): Weekly, in the evening, crewmembers filled out a brief conflict questionnaire developed for the study. They were asked to indicate, both currently and in the last 7 days, whether they had a conflict with either another crewmember or mission controllers. If they indicated a conflict had occurred, they were to indicate whether or not it was resolved. They did not have to indicate the identity of the person(s) with whom they indicated they had a conflict.

Data acquisition for psychological measures resulted in 100% completed tests (i.e., N = 6 for SDS-17; N = 444 for BDI-II; POMS-SF;CQ; VAS for workload and sleep quality ratings; and N = 888 for VAS scales for unhappiness, physical exhaustion, mental fatigue, stress, tiredness).

2.2.1.6 Post-mission debrief interviews: During individual crewmember's debrief interviews the second day post-mission confinement, crewmembers were asked to name the two crewmembers with whom they communicated most frequently.

#### 2.2.2 Behavioral measures

2.2.2.1 Actigraphy: Actigraphy is a reliable, non-invasive method to validly assess rest-activity cycles [13]. Throughout the 520-day simulated mission to Mars, each crewmember continuously wore a wristwatch size actigraph (*Actiwatch Spectrum, Philips/Respironics*) on the wrist of the non-dominant arm. The device measured both average white light intensity (illuminance in Lux) and a calibrated activity level from movement-induced accelerations of the wrist. It also displayed clock time. In the Mars 520-day study actigraphs recorded one activity and one illuminance value per minute. A validated algorithm [14] was used to automatically classify 1-min actigraphy epochs into active wake, sleep, or waking rest (Respironics Actiware, Version 5.59.0015, standard settings). In cases of obvious misclassification, the automatic scoring was corrected manually (less than 2.8% of the automatic scoring was corrected this way). Epochs with off-wrist or missing data (due to data downloads or equipment failure) were classified accordingly. Overall, 4,396,333 min (73,272 h or 98.0%) of valid actigraphy data were obtained from the 6 crewmembers while they lived in the facility throughout the Mars 520-day study. For statistical analyses off-wrist or missing actigraphy epochs were imputed with averages of non-missing epochs calculated for each crewmember, each mission quarter, and each of the 1440 min of the day.

2.2.2.2 Psychomotor Vigilance Test (PVT-B): Once per week, each crewmember performed a 3-minute version of the Psychomotor Vigilance Test on a calibrated laptop computer (*Pulsar Informatics, Inc.*) to assess the effects of potential changes in sleep-wake behavior. The PVT-B measures vigilant attention by recording response time (RT) to visual stimuli that occur at random inter-stimulus intervals (ISI), and it has negligible aptitude and learning effects [15], [16]. The brief PVT (i.e., PVT-B) was validated against the standard 10-minute PVT [17] and shown to predict performance on a simulated luggage screening task [18]. Each crewmember performed the PVT-B on a different day of the week, once in the morning after waking up and once in the evening. The test required visually monitoring a red rectangular box on the computer screen, and pressing a response button as soon as a yellow stimulus counter appeared, which stopped the counter and displayed the RT in milliseconds for a 1 s period. ISIs varied randomly from 2–5 s. Data acquisition for PVT-B resulted in N = 888 completed tests, which was 100% of the data sought.

### 2.3 Statistical analyses

To analyze time in mission effects, mixed model ANOVAs (Proc Mixed, SAS Version 9.3, SAS Institute Inc., Cary, NC) with a random intercept for crewmembers and unstructured covariance were performed with mission quarter (MQ) as the only explanatory variable (MQ1, days 1–130; MQ2, days 131–260; MQ3, days 261–390; MQ4, days 391–520) and with the scores from the mood scales (BDI-II and POMS-SF) and visual analog scales as outcome variables. Although we could have justified many different hypotheses relative to time in mission (e.g., steadily increasing or decreasing effects, third quarter effect), we chose to keep our hypothesis as generic as possible (null hypothesis: no difference between mission quarters). This was partially driven by findings on the activity data that showed a steep decline in activity initially, a slow but steady decline during the second and third mission quarters, and a sharp rise at the end of the mission, which conformed to neither of the two above-stated hypotheses [19]. Our mixed model analyses took the clustered nature of the data into account and used all available data points based on repeated measures within subjects (N = 444 for measures sampled only in the morning or in the evening and N = 888 for measures sampled both in the morning and the evening). The models for outcomes sampled both in the morning and the evening were also controlled for administration time (morning or evening). If a type 3 test indicated a significant MQ effect (P<0.05), post-hoc tests comparing each MQ with each other MQ were performed. Post-hoc tests were Bonferroni corrected for Type I error inflation (α = 0.05/6 = 0.0083).

To investigate individual differences between crewmembers, ANOVAs (Proc Mixed in SAS) were performed with crewmember as the only explanatory variable and with the scores from the mood scales (BDI-II and POMS-SF) and visual analog scales as outcome variables. Again, models with visual analog scale variables sampled twice daily were also controlled for administration time (morning or evening). If a Type 3 test indicated a significant crewmember effect (P<0.05), post-hoc tests comparing data from each crewmember with data from each of the other crewmembers were performed. Post-hoc tests were Bonferroni corrected for Type I error inflation (α = 0.05/15 = 0.0033). For ease of interpretation, all scales were transformed to a 0 to 100 range in Tables 1 and 2.

**Table 1:** Effects of time-in-mission on psychological measures.

Scales	Mission	Mission Quarter				ANOVA
	Average	1	2	3	4	P-value
**Depression**						
Beck Depression Inventory-II	2.2 (2.0)	1.5 (1.4)^3,4^	1.4 (1.4)^3,4^	3.1 (2.8)^1,2^	2.8 (2.5)^1,2^	<0.0001
**Mood**						
POMS Depression-Dejection	0.9 (0.7)	0.6 (0.4)	0.9 (0.8)	1.0 (0.8)	1.2 (1.0)	0.5078
POMS Vigor-Activty	48.3 (9.2)	49.8 (8.8)^2^	44.5 (11.3)^1,4^	48.6 (8.9)	50.5 (8.4)^2^	0.0011
POMS Confusion-Bewilderment	5.9 (2.6)	4.6 (2.5)^3,4^	4.6 (2.3)^3,4^	6.9 (3.2)^1,2^	7.7 (3.1)^1,2^	<0.0001
POMS Tension-Anxiety	1.8 (1.2)	1.6 (0.8)	4.6 (2.3)	1.9 (1.7)	2.0 (1.7)	0.9086
POMS Anger-Hostility	1.5 (1.1)	1.1 (0.6)	1.4 (1.1)	1.5 (1.2)	2.0 (1.7)	0.4169
POMS Fatigue-Inertia	3.3 (1.8)	2.6 (0.9)	2.9 (1.8)	3.8 (2.1)	3.9 (2.5)	0.1976
POMS Total Mood Disturbance Score	10.4 (2.2)	9.7 (1.8)	10.7 (2.4)	10.6 (2.4)	10.6 (2.5)	0.1413
**Visual Analog Scales**						
Happy-Unhappy	19.8 (8.0)	18.2 (6.9)	20.6 (8.6)	20.3 (8.1)	20.0 (8.4)	0.0984
Healthy-Sick	11.1 (5.6)	9.4 (4.6)^3,4^	9.1 (5.5)^3,4^	13.2 (6.7)^1,2^	12.7 (6.4)^1,2^	<0.0001
Energetic-Physically Exhausted	20.6 (6.1)	23.4 (5.5)^3,4^	20.5 (6.1)	20.4 (7.4)^1^	18.0 (6.4)^1^	<0.0001
Mentally Sharp-Mentally Fatigued	19.8 (8.5)	20.0 (7.9)	20.5 (9.1)	19.5 (8.8)	19.3 (8.5)	0.5640
Not Stressed-Very Stressed	14.3 (6.3)	12.4 (5.6)	14.6 (6.3)	15.1 (7.0)	14.9 (6.7)	0.0301
Ready to Go-Tired	25.4 (6.8)	23.5 (4.9)^2^	27.3 (7.4)^1^	26.3 (7.7)	24.3 (7.5)	0.0173
Good Sleep Quality-Poor Sleep Quality	22.9 (7.1)	25.1 (5.9)	24.1(7.6)	21.7 (7.7)	20.4 (8.2)	0.1531
Low Workload-High Workload	34.8 (5.1)	43.2 (4.7)^2,3,4^	33.4 (5.8)^1^	30.3 (4.6)^1^	32.0 (8.0)^1^	<0.0001

**Table 1** **:** Superscript number 1–4 indicate a significant difference to the respective mission quarter at α = 0.05 after Bonferroni correction of post-hoc tests. All scales were transformed to a 0 to 100 range. A score of 100 represents the maximal expression (e.g., maximal depression, maximal tension-anxiety, maximal unhappiness, etc.). Standard errors are reported in parenthesis. P-value for the main effect of mission quarter is reported in the last column. POMS: Profile of Mood States Short Form.

**Table 2:** Inter-individual differences in psychological measures.

Scales	Crewmember							ANOVA
	a	b	c	d	e	f	ICC	P-value
**Social Desirability**								
Social Desirability Scale-17	81	69	56	56	25	81		
**Depression**								
Beck Depression Inventory-II	0^e,^ *	0^e,^ *	0^e,^ *	0.4 (0.1)^e^	12.1 (0.9)^a,b,c,d,f^	0.5 (0.1)^e^	0.679	<0.0001
**Mood**								
POMS Depression-Dejection	0.0 (0.0)^e^	0.0 (0.0)^e^	0.1 (0.1)^e^	0.1 (0.1)^e^	4.6 (0.8)^a,b,c,d,f^	0.8 (0.3)^e^	0.251	<0.0001
POMS Vigor-Activty	41.3 (2.4)^b,c,d,e,f^	48.8 (0.7)^a,c,d,e,f^	77.0 (0.7)^a,b,e,f^	72.2 (1.0)^a,b,e,f^	22.0 (1.4)^a,b,c,d,f^	28.5 (1.6)^a,b,c,d,e^	0.772	<0.0001
POMS Confusion-Bewilderment	0^b,c,e,^ *	13.2 (0.3)^a,c,d,f^	6.8 (0.8)^b,d,e,f^	0.5 (0.2)^b,c,e^	13.8 (0.9)^a,c,d,f^	1.4 (0.5)^b,c,e^	0.632	<0.0001
POMS Tension-Anxiety	0^e,^ *	0^e,^ *	0.9 (0.4)^e^	0.7 (0.3)^e^	7.5 (1.0)^a,b,c,d,f^	1.7 (0.5)^e^	0.322	<0.0001
POMS Anger-Hostility	0.1 (0.1)^e^	0^e,^ *	0.2 (0.1)^e^	0.4 (0.3)^e^	6.7 (1.0)^a,b,c,d,f^	1.9 (0.5)^e^	0.293	<0.0001
POMS Fatigue-Inertia	1.3 (0.5)^e^	0.2 (0.2)^c,e^	3.2 (0.6)^b,e^	1.8 (0.6)^e^	12.0 (1.2)^a,b,c,d,f^	1.4 (0.4)^e^	0.376	<0.0001
POMS Total Mood Disturbance Score	9.7 (0.4)^c,d,e,f^	10.1 (0.1)^c,d,e,f^	5.3 (0.3)^a,b,e,f^	5.0 (0.3)^a,b,e,f^	19.6 (0.8)^a,b,c,d,f^	12.8 (0.3)^a,b,c,d,e^	0.701	<0.0001
**Visual Analog**								
Happy-Unhappy	0.5 (0.5)^b,c,e,f^	9.8 (0.3)^a,c,d,e,f^	20.9 (0.7)^a,b,d,e,f^	2.6 (1.0)^b,c,e,f^	49.8 (1.4)^a,b,c,d,f^	35.1 (1.1)^a,b,c,d,e^	0.753	<0.0001
Healthy-Sick	0.0 (0.0)^c,e,f^	0.5 (0.3)^c,e,f^	15.2 (0.5)^a,b,d,e^	0.7 (0.5)^c,e,f^	35.2 (1.2)^a,b,c,d,f^	14.8 (1.3)^a,b,d,f^	0.671	<0.0001
Energetic-Physically Exhausted	9.1 (1.2)^c,d,e,f^	11.4 (0.3)^c,d,e,f^	23.6 (0.8)^a,b,d,e,f^	3.6 (0.7)^a,b,c,e,f^	41.3 (1.2)^a,b,c,d,f^	34.3 (1.6)^a,b,c,d,e^	0.587	<0.0001
Mentally Sharp-Mentally Fatigued	0.8 (0.7)^c,e,f^	1.5 (0.7)^c,e,f^	31.8 (0.6)^a,b,d,e,f^	1.0 (0.5)^c,e,f^	44.1 (1.3)^a,b,c,d,f^	40.0 (1.2)^a,b,c,d,e^	0.788	<0.0001
Not Stressed-Very Stressed	0.0 (0.0)^c,e,f^	0.3 (0.1)^c,e,f^	21.5 (0.8)^a,b,d,e,f^	1.1 (0.6)^c,e,f^	31.1 (1.3)^a,b,c,d^	31.5 (1.5)^a,b,c,d^	0.669	<0.0001
Ready to Go-Tired	10.1 (1.4)^c,e,f^	12.2 (0.3)^c,e,f^	41.8 (1.1)^a,b,d^	8.7 (0.9)^c,e,f^	37.2 (1.6)^a,b,d,f^	42.3 (1.4)^a,b,d,e^	0.563	<0.0001
Good Sleep Quality-Poor Sleep Quality	9.2 (3.1)^c,e,f^	12.3 (1.1)^c,e,f^	22.2 (1.5)^a,b,d,e,f^	5.4 (1.6)^c,e,f^	41.1 (2.0)^a,b,c,d^	47.3 (2.1)^a,b,c,d^	0.508	<0.0001
Low Workload-High Workload	30.1 (4.1)^c,d,e,f^	23.8 (1.7)^c,e,f^	42.7 (2.1)^a,b,d^	18.5 (2.9)^a,c,e,f^	43.2 (2.4)^a,b,d^	50.3 (2.7)^a,b,d^	0.211	<0.0001

**Table 2** **:** Superscript letters a–f indicate a significant difference to the respective crewmember at α = 0.05 after Bonferroni correction for post-hoc tests. All scales were transformed to a 0 to 100 range. A score of 100 represents the maximal expression (e.g., maximal depression, maximal tension-anxiety, maximal unhappiness). Standard errors are reported in parenthesis. P-value for the main effect of crewmember is reported in the last column. POMS: Profile of Mood States Short Form; ICC: Intra-class Correlation;

* indicates that the respective crewmember answered 0 on all items throughout the whole mission.

To investigate changes of individual differences with time in mission, graphs plotting cumulative scores of mood and visual analog scale outcomes relative to time in mission were generated for those variables with a significant (P<0.05) main effect for mission quarter. To further investigate individual differences, we calculated intra-class correlations (ICC) for each outcome measure as the ratio of between-subjects variance to the sum of the between- and within-subjects variances. The ICC is based on variance components analysis, involving the explicit separation of within-subjects variance and between-subjects variance in data derived from repeated measurements in individuals. The ICC expresses the proportion of variance in these data that is explained by systematic inter-individual variability. Stability of ICC values was interpreted using the following ranges: “slight” (0.0–0.2); “fair” (0.2–0.4); “moderate” (0.4–0.6); “substantial” (0.6–0.8); and “almost perfect” (0.8–1.0) [20]. We compared actigraphy scorings across subjects on a minute per minute basis. One minute epochs that were classified as missing or off-wrist for at least one crewmember were excluded from the analysis (86,068 min or 11.5% of the 520-day period). For each crewmember, those minutes were counted where the crewmember was either the only crewmember sleeping or the only crewmember active awake. We then calculated the cumulative time for both categories corrected for the amount of missing data (i.e., relative to the full 520-day or 12,480-h mission).

## Results

### 3.1 Psychological measures

Table 1 reports the psychological measures as averages across all crewmembers for the whole mission and for individual mission quarters; whereas Table 2 shows psychological measure averages across the whole mission for each individual crewmember and also provides information on ICCs as an indicator of the degree of systematic inter-individual variability in self-report outcomes.

**Figure 1:**
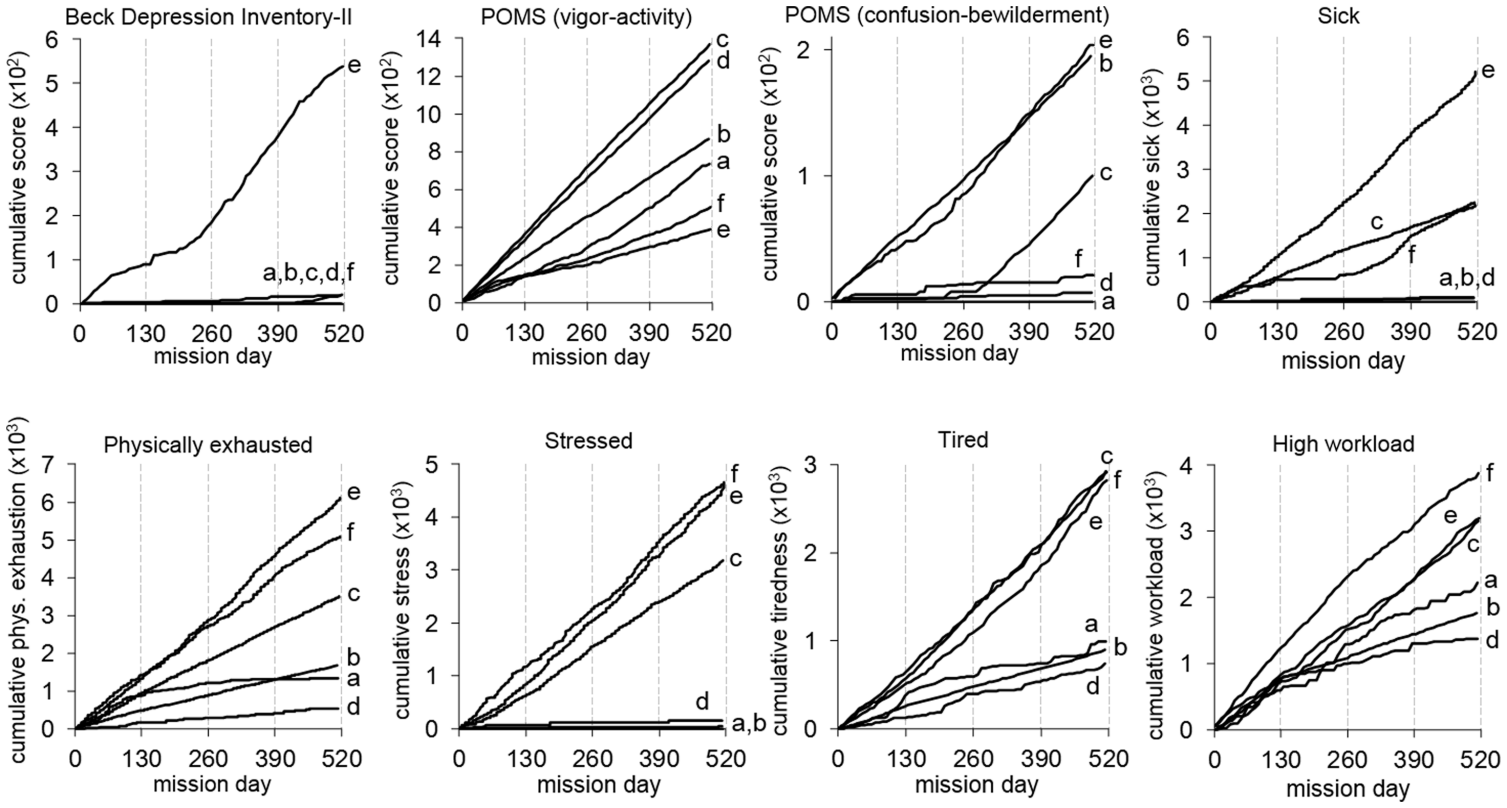
Cumulative self report scores show differential reactions to confinement. Cumulative scores are plotted for each crewmember (identified by lower case letters *a–f*) relative to time in mission for the 8 self-report measures that showed significant differences between crewmembers (see Table 2). Beck-Depression Inventory-II and Profile of Mood States Short Form (POMS) scores were not transformed to a range from 0 to 100 for this figure.

At the level of the whole crew and for the whole mission, the average BDI-II score of 2.2 out of 100 indicated no depression among crewmembers. Although BDI-II scores were significantly higher in the second compared to the first half of the mission, they were very low at both mission phases (3.1 and 2.8 out of 100, respectively). Crewmember *e* was the only crewmember to report symptoms of depression (Figure 1a). Even with the suicide question removed, his BDI-II score indicated a mild depression in 7 out of 74 weeks (9.5% of mission time) and a moderate depression in 1 out of 74 weeks (1.4% of mission time). The reported symptoms more often included somatic (58.2%) rather than affective (41.8%) symptoms, with the top 5 items being “changes in sleep patterns” (9.5%), “punishment feelings” (8.7%), “tiredness and/or fatigue” (8.2%), “guilt feelings” (8.0%), and “loss of pleasure” (8.0%). Crewmember *e* also had the lowest per-mission social desirability scale score than the rest of the crewmembers (Table 2). In contrast to crewmember *e*, crewmembers *a*, *b*, and *c* checked off none of the 20 items during all 74 administrations of the BDI-II indicating no depressive symptoms.

Similar to the BDI-II findings, the average POMS-SF score for the crew and for the whole mission showed no consistent signs of elevation in total mood disturbance (score of 10.4 out of 100) or psychological distress on any of the subscales (scores ranging from 0.9 to 5.9 out of 100). Crewmembers indicated a medium level of vigor and activity throughout the mission (48.3 out of 100). Significant changes between mission quarters were only observed for vigor-activity (P = 0.0011), which was lowest in mission quarter 2, and confusion-bewilderment (P<0.0001), which was higher in the second compared to the first half of the mission (Table 1 and Figure 1). Again, crewmember *e* scored highest on total mood disturbance and all subscales of the POMS, except for vigor-activity where he had the lowest score (22.0 out of 100).

At the level of the whole crew and for the whole mission, visual analog scale ratings indicated low levels (range 11.1–25.4 out of 100) of feeling unhappy, sick, physically exhausted, mentally fatigued, stressed, or tired (Table 1). Crewmembers had stronger feelings of sickness in the second compared to the first half of the mission, and the reported tiredness was maximal in the second mission quarter. Sleep quality was rated on average as good (22.9 out of 100) and showed no reliable changes with time in mission. Workload was rated low to medium (34.8 out of 100) for the mission, but it was perceived as significantly higher in the first quarter compared to subsequent mission quarters. Feelings of being unhappy, sick, physically exhausted, or mentally fatigued were rated highest by crewmember *e*, whereas crewmember *f* indicated the highest levels for stress, tiredness, poor sleep quality, and high workload (Table 2, Figure 1). He was also the crewmember who averaged the lowest sleep time across the mission (see above).

We found significant inter-individual differences for all self-report measures at P<0.0001 (Table 2). Intra-class correlations (ICC) and cumulative functions (Figure 1) were used to determine if these individual reactions were stable during the mission and potentially phenotypic. ICCs indicated substantial stability of individual differences during the mission in depression inventory scores (0.679), POMS ratings of vigor-activity (0.772), confusion-bewilderment (0.632), and total mood disturbance (0.701), and visual analog scale ratings of unhappiness (0.753), sickness (0.671), mental fatigue (0.788), and stress (0.669). On average, more than half (55%) of the variance in self-report outcomes was attributable to stable differences among crewmembers. Cumulative functions also suggest substantial trait-like consistency throughout the mission confinement. For example, crewmember *d* consistently had among the highest vigor-activity ratings (POMS-SF) and lowest physical exhaustion (VAS), stress (VAS), and tiredness ratings (VAS); while crewmembers *e* and *f* had the lowest vigor-activity ratings, and the highest physical exhaustion, stress and tiredness ratings across the mission; and crewmember *c* showed a mixed pattern of high vigor-activity, moderate physical exhaustion and stress, and high tiredness across the mission (Figure 1).

**Figure 2:**
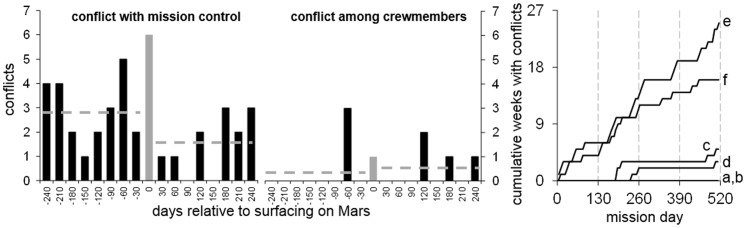
Perceived conflicts throughout the simulated Mars mission. The number of conflicts with mission control (left panel) and other crewmembers (middle panel) were counted for 30-day periods relative to a 30-day period surrounding the landing on Mars between mission days 244 and 273. One conflict was counted if the crewmember recorded either a current conflict and/or a conflict in the past seven days. Conflicts (reported once weekly) with mission control peaked during the Mars landing period, were lower in the second half compared to the first half of the mission (dashed lines represent averages over pre- and post-landing periods), and were reported more often than conflicts among crewmembers. The right panel shows the cumulative number of weeks with conflicts relative to time in mission by crewmember. The majority of conflicts were reported by crewmembers *e* and *f*.

Figure 2 summarizes the results of the conflict questionnaire. Crew-reported conflicts with mission control peaked during the 30 days of Mars surfacing, were higher in the first half compared to the second half of the mission (23∶12), and were reported 5 times more often than conflicts among crewmembers (41∶8). Two crewmembers (*e*, *f*) reported the majority (85%) of the conflicts (51% and 34%, respectively).

**Figure 3:**
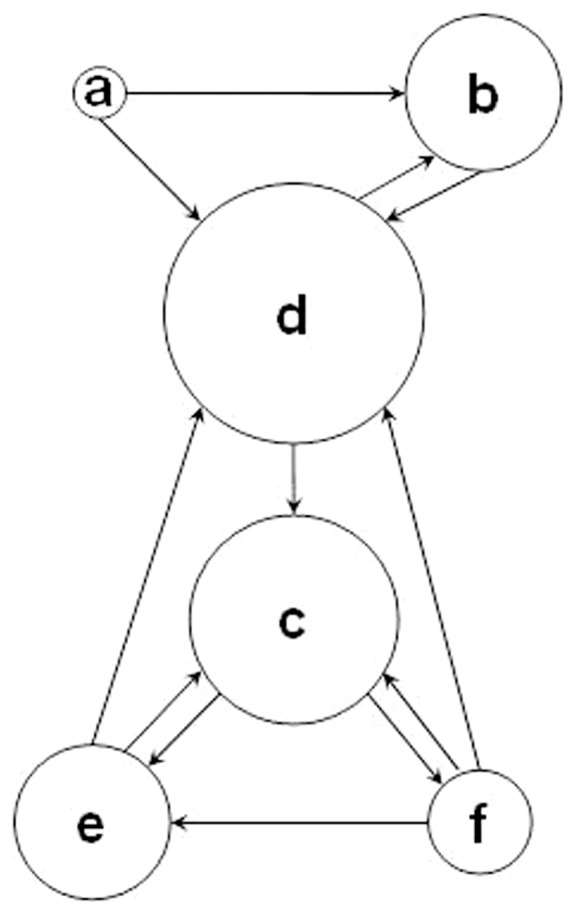
Crew interactions were facilitated by a core group. During de-briefs, each crewmember was asked to report the two crewmembers interacted with most frequently during the mission. Arrows pointing to a crewmember indicate the number of times he was mentioned by others; those pointing away designate with whom he indicated he most often interacted. Circle size indicates the frequency with which a crewmember was identified as interacted with most frequently.

During crew debriefs after the mission, crewmembers were asked to name the two crewmembers with whom they communicated most frequently throughout the 520-day of confinement. The answers to this question are depicted in Figure 3. Based on frequency of all crewmembers' responses, crewmembers *d* and *c* had a central role in team communication. In contrast, crewmember *a* was not mentioned by any other crewmember, and crewmember *f* was only mentioned by one other crewmember.

## Discussion

IBMP's historically long, and behaviorally realistic, 520-day simulated mission to Mars involved all the anticipated features of isolation and confinement required for exploration space missions [19]. The unprecedented duration, high fidelity, and ecologic validity of the simulation make the Mars-520 study unique and in many ways superior to a number of space simulations performed in the past [1]–[3], [22], [23].

This manuscript focuses (a) on a description of the Mars 520 mission crews' subjective ratings of mood, psychological distress, health, stress, fatigue, sleep quality, and workload, (b) on changes in behavior and psychological state with time in mission, and (c) on differences between individual crewmembers. Results on changes in sleep-wake timing, movement activity, and psychomotor vigilance performance have been reported in detail elsewhere [19] and shall only be briefly summarized here for contextual interpretation of the psychological data.

Sleep time averaged 7.39 h (SE = 0.20) per 24 h across the mission for all crewmembers, ranging from 6.54 h (crewmember *f*) to 7.94 h (crewmember *a*) between crewmembers, and increased with time in mission. Crewmember *f*, who averaged the highest stress ratings and worst sleep quality ratings during the mission (Table 2), was the only crewmember whose sleep time decreased during the mission due to a worsening sleep onset insomnia. He was also the only crewmember with impaired PVT-B performance and accounted for the majority of errors of omission (i.e., lapses = 64%) and errors of commission (i.e., false starts = 48%). Crewmember *d* was by a factor of 1.85 more active while awake than the rest of the crew, but still averaged the second longest sleep time across the mission (7.79 h). Crewmember *a* manifested a split-sleep pattern (i.e., nocturnal anchor sleep plus a diurnal nap) during the mission, while crewmember *b* was behaviorally free-running with a dominant period of ca. 25 h. These two crewmembers (*a, b*) were asleep when the other crewmembers were awake (or vice versa) during a total of 20.1% of the mission. The other four crewmembers (*c, d, e, f*) had a monophasic nocturnal sleep pattern with a 24 h sleep-wake cycle throughout the mission [19].

A progressive sedentariness of the crew was evident through increased sleep time and decreased workload ratings with time in mission [19]. This highlights the need for coping strategies that address monotony and boredom from low workload after the first mission quarter, when communication delays with mission control became pronounced [24]. Coping strategies will also be needed for hypostimulation and restricted social contacts during long-duration missions [2].

A modest increase in depressive symptoms and psychological distress was observed in the second compared to the first half of the mission, but this effect was largely contributed to by crewmember *e*. A higher frequency of crew-perceived conflicts with mission control was reported in the first relative to the second half of the mission (being maximal during the period of the simulated landing on Mars). According to Shved et al. [25], both the number of crew interactions (overall amount of communication) with mission control and the number of negative and critical statements in crew messages increased during the simulated landing period. We did not find a third quarter effect [26] in any of the psychological or behavioral outcomes. The fact that conflicts with mission control were reported by crewmembers five times more often than conflicts among themselves highlights the importance of a good relationship between the crew and mission controllers and the need for a greater involvement of mission controllers in pre-mission training, as has been noted by others [27]. Additionally, greater crew autonomy might reduce conflicts between the crew and mission control.The 520-day simulated Mars mission was completed without any of the crewmembers discontinuing the study prematurely. Moreover, our data and debriefs of the crew data revealed no signs of major behavioral emergencies or serious unresolved conflicts during the mission. This overall mission success is reflected in average scores across crewmembers for many of our outcomes (e.g., sufficiently long sleep, high levels of psychomotor vigilance performance, no indication of depression, low levels of psychologic distress, high ratings of happiness, health, energy, and low ratings of stress, mental fatigue, and tiredness). These results may have been the effect of the psychological support the crew received throughout the mission [24]. However, such findings do not indicate the mission was without behavioral distress for individual crewmembers, as our results also indicated stable inter-individual differences among crewmembers for practically all behavioral health outcomes. This finding is in contrast with an earlier isolation study that was performed at IBMP in Moscow (SFINCSS-99) and included 3 crews of 4 crewmembers each that were confined for 240 days (group 1, 4 Russians) and 110 days (group 2, 1 German and 3 Russians; and group 3, 1 Russian, 1 Austrian, 1 Japanese, and 1 Canadian). Group 3 entered and shared the facility with group 1 after the study ended for group 2. The crew was all male except for one female crewmember. One crewmember of group 3 discontinued the study prematurely on mission day 63, likely as a consequence of a conflict between crewmembers at a New Year's celebration [2], [3]. In contrast to Mars 520, the 3 groups involved in SFINCSS-99 did not know each other and did not perform joint training prior to the mission.

There were many examples of inter-crew differences in coping with the prolonged isolation and confinement of the 17-month high-fidelity mission. Crewmember *b* was behaviorally free-running with a dominant period of 24.98 h, and thus his sleep was approximately equally distributed over the 24-h day throughout the mission [19]. Crewmember *a* manifested a split-sleep pattern with frequent naps during the day that lengthened towards the end of the study. As a consequence, crewmembers *a* and *b* would have been at risk for performing suboptimal on mission tasks that were scheduled during the daytime. Also, as both crewmembers were frequently sleeping when the rest of the crew was awake (and vice versa), the time for interaction with the rest of the crew was also reduced [19], which is probably one reason for the lower frequency at which crewmembers *a* and *b* were mentioned by other crewmembers relative to frequency of communication (Figure 3). Crewmember *f* had the lowest average sleep time in mission (6.54 h), and the highest mission average ratings of tiredness, physical exhaustion, stress and poor sleep quality [19]. The sleep-wake data indicated crewmember *f* experienced a worsening sleep onset insomnia across the mission, which resulted in his being the only crewmember averaging less than 7 hours sleep a day in the across the mission [19]. Six or fewer hours of sleep a day on a chronic basis has been shown to lead to escalating errors in psychomotor vigilance performance [28]–[30]. This was the case for crewmember *f*, who had the majority of PVT-B errors of omission and commission among the crew. This degradation of behavioral alertness could be detrimental during critical periods of the mission (e.g., docking maneuvers, extra-vehicular activities, or emergencies).

Crewmember *e* was the only crewmember to frequently report symptoms of depression that increased during the second half of the mission. He also had the highest ratings of psychological distress and of feeling unhappy, sick, physically exhausted and mentally fatigued. Although crewmember *e* was the only subject to report these symptoms, it is unclear whether he was the only subject that experienced them, as the other subjects showed much higher social desirability bias scores (SDS-17) compared to crewmember *e*. Thus, crewmember *e* had the lowest pre-mission bias in presenting himself ideally, while some other crewmembers (e.g., *a* and *f*) had much higher SDS-17 scores indicating a tendency to present themselves more ideally. This bias may have resulted in their misreporting negative symptoms during the mission. This reporting bias could also be based in cultural differences among crewmembers [31]. Crewmember *e* (together with crewmember *f*) reported most of the conflicts with mission control and other crewmembers. Comparable to crewmembers *a* and *b*, crewmembers *e* and *f* had a lower frequency at which other crewmembers mentioned them relative to frequency of communication (Figure 3). In contrast, crewmembers *c* and *d* were notable for showing no signs of behavioral changes or psychological distress during the mission; they were most often mentioned as the two people with whom the rest of the crew interacted; and they were the only two crewmembers to suffer no changes in sleep duration, sleep-wake timing or sleep quality during the 520-day mission.

When all Mars 520 behavioral and psychological data are considered in aggregate, only two of the six crewmembers (*c* and *d*) showed neither behavioral disturbances nor reports of psychological distress during the 17-month period of mission confinement. This meta- finding highlights the importance of identifying behavioral, psychological, and biological markers of the characteristics that predispose prospective long-duration space exploration crewmembers to both effective and ineffective neurobehavioral and psychosocial reactions to the prolonged confinement required for exploration missions. Such predictors and biomarkers are needed to inform crew selection, training, and individualized countermeasures. This conclusion and the findings of this study are consistent with recent reviews of the psychological effects of polar expeditions and other analogs for space flight [2], [32], [33], [34].

The age of exploration space missions will require the “right stuff” for prolonged confinement and isolation, which the Mars 520 ICE experiment indicates means good insight into one's capability, behavioral health, biological adaptability, environmental coping, mental endurance, and salutogenic responses to stressors [35]. This conclusion is not only consistent with findings from polar research as a space analog [21], [33], [34], [36], but they should also be priorities in crew selection and training in confined environments for the mission to Mars and beyond.

Finally, we note that the vast majority of both adequate and inadequate psychological and behavioral reactions we observed in Mars 520 crewmembers appeared to be phenotypic (as evidenced by high ICCs, Table 2). Moreover, they appeared relatively early in the mission and sustained unabated throughout it. It suggests that it may be possible to detect individual psychological and behavioral vulnerabilities in periods that are significantly shorter than the 520 days employed in the IBMP study. This would enhance capability to efficiently select and train crew before, and monitor and provide them with adequate, individualized countermeasures during a long-duration mission.

## Limitations

This study has several limitations. Naturally, microgravity, radiation and threat-to-life–, three important physiological and psychological stressors that will be encountered during exploration-type missions– could not be simulated in Mars 520, which restricts the generalizability of the findings to long-duration space missions [37]. We only had limited access to the crew before and after the 520-day mission, and thus cannot infer about their psychological status before and after the mission. The medical and psychological selection and screening of the crew was conducted by the space agency responsible for each study participant, making it uncertain to what extent it was comparable. The crew was male only, so we cannot make inferences about female only or mixed crews. Our assessment of performance was limited to psychomotor vigilance testing. It cannot therefore be assumed that other aspects of cognitive performance were not changed across time in mission. We want to stress that we did not measure physiological or endocrine markers of stress, limiting our ability to detect stress reactions not revealed in the behavioral responses of crewmembers. Finally, our protocol was one of at least 90 other protocols carried out in the quasi-operational environment of the 520-day Mars mission simulation. We had no control over the other protocols that may have introduced unexplained variance in our outcome measures.
